# Inhibition of phosphatidylinositol 3-kinase catalytic subunit alpha by miR-203a-3p reduces hypertrophic scar formation via phosphatidylinositol 3-kinase/AKT/mTOR signaling pathway

**DOI:** 10.1093/burnst/tkad048

**Published:** 2024-01-02

**Authors:** Shixin Zhao, Hengdeng Liu, Hanwen Wang, Xuefeng He, Jinming Tang, Shaohai Qi, Ronghua Yang, Julin Xie

**Affiliations:** Department of Burns, The First Affiliated Hospital of Sun Yat-Sen University, No. 58 Zhongshan Second Road, Yuexiu District, Guangzhou, Guangdong, 510062, China; Guangdong Provincial Engineering Technology Research Center of Burn and Wound Accurate Diagnosis and Treatment Key Technology and Series of Products, Sun Yat-Sen University, No. 58 Zhongshan Second Road, Yuexiu District, Guangzhou, Guangdong, 510062, China; Department of Burns, The First Affiliated Hospital of Sun Yat-Sen University, No. 58 Zhongshan Second Road, Yuexiu District, Guangzhou, Guangdong, 510062, China; Guangdong Provincial Engineering Technology Research Center of Burn and Wound Accurate Diagnosis and Treatment Key Technology and Series of Products, Sun Yat-Sen University, No. 58 Zhongshan Second Road, Yuexiu District, Guangzhou, Guangdong, 510062, China; Department of Burns, The First Affiliated Hospital of Sun Yat-Sen University, No. 58 Zhongshan Second Road, Yuexiu District, Guangzhou, Guangdong, 510062, China; Guangdong Provincial Engineering Technology Research Center of Burn and Wound Accurate Diagnosis and Treatment Key Technology and Series of Products, Sun Yat-Sen University, No. 58 Zhongshan Second Road, Yuexiu District, Guangzhou, Guangdong, 510062, China; Department of Burns, The First Affiliated Hospital of Sun Yat-Sen University, No. 58 Zhongshan Second Road, Yuexiu District, Guangzhou, Guangdong, 510062, China; Guangdong Provincial Engineering Technology Research Center of Burn and Wound Accurate Diagnosis and Treatment Key Technology and Series of Products, Sun Yat-Sen University, No. 58 Zhongshan Second Road, Yuexiu District, Guangzhou, Guangdong, 510062, China; Department of Burns, The First Affiliated Hospital of Sun Yat-Sen University, No. 58 Zhongshan Second Road, Yuexiu District, Guangzhou, Guangdong, 510062, China; Guangdong Provincial Engineering Technology Research Center of Burn and Wound Accurate Diagnosis and Treatment Key Technology and Series of Products, Sun Yat-Sen University, No. 58 Zhongshan Second Road, Yuexiu District, Guangzhou, Guangdong, 510062, China; Department of Burns, The First Affiliated Hospital of Sun Yat-Sen University, No. 58 Zhongshan Second Road, Yuexiu District, Guangzhou, Guangdong, 510062, China; Guangdong Provincial Engineering Technology Research Center of Burn and Wound Accurate Diagnosis and Treatment Key Technology and Series of Products, Sun Yat-Sen University, No. 58 Zhongshan Second Road, Yuexiu District, Guangzhou, Guangdong, 510062, China; Department of Burn and Plastic Surgery, Guangzhou First People's Hospital, South China University of technology, No. 1 Panfu Road, Yuexiu District, Guangzhou, Guangdong, 510062, China; Department of Burns, The First Affiliated Hospital of Sun Yat-Sen University, No. 58 Zhongshan Second Road, Yuexiu District, Guangzhou, Guangdong, 510062, China; Guangdong Provincial Engineering Technology Research Center of Burn and Wound Accurate Diagnosis and Treatment Key Technology and Series of Products, Sun Yat-Sen University, No. 58 Zhongshan Second Road, Yuexiu District, Guangzhou, Guangdong, 510062, China

**Keywords:** Fibrosis, Hypertrophic scar, miRNA, Phosphatidylinositol 3-kinase catalytic subunit alpha, Transdifferentiation, miR-203a-3p, Phosphatidylinositol 3-kinase, AKT, mTOR

## Abstract

**Background:**

Hypertrophic scar (HS) is a common fibroproliferative skin disease that currently has no truly effective therapy. Given the importance of phosphatidylinositol 3-kinase catalytic subunit alpha (PIK3CA) in hypertrophic scar formation, the development of therapeutic strategies for endogenous inhibitors against PIK3CA is of great interest. Here, we explored the molecular mechanisms underlying the protective effects of miR-203a-3p (PIK3CA inhibitor) against excessive scar.

**Methods:**

Bioinformatic analysis, immunohistochemistry, immunofluorescence, miRNA screening and fluorescence *in situ* hybridization assays were used to identify the possible pathways and target molecules mediating HS formation. A series of *in vitro* and *in vivo* experiments were used to clarify the role of PIK3CA and miR-203a-3p in HS. Mechanistically, transcriptomic sequencing, immunoblotting, dual-luciferase assay and rescue experiments were executed.

**Results:**

Herein, we found that PIK3CA and the phosphatidylinositol 3-kinase (PI3K)/AKT/mTOR pathway were upregulated in scar tissues and positively correlated with fibrosis. We then identified miR-203a-3p as the most suitable endogenous inhibitor of PIK3CA. miR-203a-3p suppressed the proliferation, migration, collagen synthesis and contractility as well as the transdifferentiation of fibroblasts into myofibroblasts *in vitro*, and improved the morphology and histology of scars *in vivo*. Mechanistically, miR-203a-3p attenuated fibrosis by inactivating the PI3K/AKT/mTOR pathway by directly targeting PIK3CA.

**Conclusions:**

PIK3CA and the PI3K/AKT/mTOR pathway are actively involved in scar fibrosis and miR-203a-3p might serve as a potential strategy for hypertrophic scar therapy through targeting PIK3CA and inactivating the PI3K/AKT/mTOR pathway.

Highlights

- Hyper-activation of the PI3K/AKT/mTOR signaling pathway and overexpression of PIK3CA are the potential etiological factors of hypertrophic scar.
- Overexpression of PIK3CA in fibroblasts promotes cell proliferation, migration and collagen production.
- miR-203a-3p exerts an anti-fibrotic effect *in vitro* and improves HS appearance *in vivo* by inhibiting the PI3K/AKT/mTOR pathway through directly targeting PIK3CA.

## Background

Hypertrophic scar (HS) is a fibroproliferative skin disease after cutaneous injury, with loss of elasticity and poor appearance presenting physical and psychological barriers to a return to normal life [1,2]. Currently, numerous therapeutic strategies have been developed to inhibit HS formation, but few have achieved satisfactory results [3]. Patients with severe HS often undergo multiple surgeries and adjunct interventions to improve scar appearance and function. Consequently, further elucidation of the molecular mechanisms underlying HS formation will contribute to the development of novel prophylactic and therapeutic strategies.

Phosphatidylinositol 3-kinase (PI3K) is composed of a regulatory subunit (p85) and a catalytic subunit (p110) [4]. The protein encoded by PIK3CA represents the catalytic subunit, which has been implicated in activating the PI3K/AKT/mTOR signaling pathway [5,6]. PIK3CA has been proven to be an oncogene due to its potent ability to promote tumor cell proliferation [7,8]. Beyond its role in malignancies, emerging research suggests that PIK3CA also plays a critical role in benign fibrotic diseases: it has been observed that PIK3CA drives prostatic stromal hypertrophy and collagen accumulation in a mouse model, while PIK3CA deficiency reduces myocardial fibrosis by inhibiting the PI3K/AKT signaling pathway [9,10]. In addition, PIK3CA has been reported as a key regulator involved in excessive scarring [11]. Thus, finding endogenous inhibitors of PIK3CA has important implications for HS mitigation.

MicroRNAs, as epigenetic regulators, can regulate fibrosis by causing targeted mRNA degradation or translational suppression [12]. It has been verified that overexpression of miR-203 inhibits fibrosis in diabetic hearts, while miR-203 blockade is confirmed to promote lung-fibroblast proliferation [13,14]. Our bioinformatic analysis identified that miR-203a-3p possesses PIK3CA 3′ untranslated region (UTR) binding sites. As noted above, we postulated that miR-203a-3p might exert an antifibrotic effect by targeting PIK3CA in HS.

In this study, we demonstrated that PIK3CA was overexpressed in both human hypertrophic scar (HHS) and rat hypertrophic scar (RHS) tissues, and proved a positive correlation between PIK3CA and fibrosis. We then screened out miR-203a-3p as a potential inhibitor of PIK3CA. The *in vitro* experiments illustrated that miR-203a-3p suppressed the proliferation, migration, collagen synthesis and contractility, as well as transdifferentiation of fibroblasts into myofibroblasts. Moreover, *in vivo* experiments showed that miR-203a-3p improved HS appearance by reducing collagen deposition and inhibiting the transdifferentiation of fibroblasts into myofibroblasts. Furthermore, we provided the evidence that miR-203a-3p attenuated HS formation by inactivating the PI3K/AKT/mTOR pathway by targeting PIK3CA.

## Methods

### Data retrieval and processing

RNA sequencing data for three HHS tissues and three HNS tissues were retrieved from the Gene Expression Omnibus (GEO) database (GSE181540) [15]. First, samples were compared by principal component analysis (PCA) and hierarchical clustering analysis. Second, differentially expressed genes (DEGs) were analyzed between HHS and HNS using the R package ‘limma’, and the screening threshold was set as |log fold change (FC) | ≥ 1 and adjusted *p* value <0.05. Volcano plots were generated using the ‘ggplot2’ R package. Third, the ‘clusterProfiler’ and ‘ggplot2’ R packages were utilized to analyze and visualize the functions of the identified DEGs, including Gene Ontology (GO), Kyoto Encyclopedia of Genes and Genomes (KEGG) and gene set enrichment analysis (GSEA). Thereafter, a Venn diagram was applied to illustrate the intersection of DEGs and PI3K/AKT/mTOR pathway-related genes. Finally, the expression profiles of 13 candidate genes were visualized in heatmap format.

### Patients and samples

Eight paired HHS tissues and adjacent HNS tissues were obtained from surgical specimens from patients undergoing HS reconstruction (Table S1, see online supplementary material). Eight paired samples were collected and divided into three parts: one was paraffin-embedded for immunohistology, the second was isolated to obtain primary fibroblasts and the third was used for RNA and protein extraction. This study followed the Helsinki Declaration and received approval from the Ethics Committee Board of the First Affiliated Hospital of Sun Yat-Sen University. Written informed consent was obtained from all participants.

### Animal model

Male Sprague–Dawley rats aged 8 weeks were purchased from the Yaokang Biotechnology Co. Ltd (Guangzhou, China). All experimental procedures were approved by the Ethics Committee Board of the First Affiliated Hospital of Sun Yat-Sen University and performed in accordance with the NIH Guide for the Care and Use of Laboratory Animals. Briefly, an 8 × 8 mm full-thickness skin defect was created on the dorsal side of the rat tail. Then the wound site was stretched by attaching a 2 cm stainless steel ring to the ventral side of the tail. After 3 weeks, RHS had been successfully epidermalized. Thereafter, 24 rats were randomly assigned into two groups: the miR-203a-3p overexpression group (Lv-miR-203a-3p) and the control group (Lv-control). We administered weekly subcutaneous injections of Lv-control or Lv-miR-203a-3p to the scar. On day 28, all rats were sacrificed and the scar tissues were surgically removed for experiments.

### Histology assay

Tissue samples were embedded and sliced after being fixed in 4% paraformaldehyde. Then the sections were stained with hematoxylin and eosin (H&E), Masson’s trichrome and picrosirius red after deparaffinization and rehydration. For the measurement of the collagen ratio, the picrosirius red-stained sections were observed with polarized light in addition to bright light.

### Immunohistochemistry assay

Sections were deparaffinized, rehydrated, washed and then incubated with the primary antibody against PIK3CA overnight at 4 °C, followed by incubation with biotinylated secondary antibodies for 30 min at 37 °C. A further 30 min of incubation with horseradish peroxidase-coupled streptavidin followed by diaminobenzidine (DAB) staining was performed on the sections. Images were collected by a fully automatic pathological section scanner (KF-PRO-020, Ningbo, China). The average optical density of PIK3CA was analyzed using ImageJ software. The antibodies used are specified in Table S2, see online supplementary material.

### Immunofluorescence assay

Cell samples were fixed in 4% paraformaldehyde for 15 min at room temperature. Both the cell samples and tissue sections were then washed, permeabilized and blocked. We used corresponding antibodies to investigate protein expression. Images were captured using a confocal microscope (Zeiss, Oberkochen, Germany). Antibody information is listed in Table S2, see online supplementary material.

### Quantitative real-time PCR assay

TRIzol reagent (Invitrogen, California, USA) was used to extract total RNA from HS tissues or cell samples. Then cDNA was synthesized using RevertAid Reverse Transcriptase (Thermo Fisher Scientific, Waltham, USA). mRNA expression levels were quantified using a SYBR Green Real-time PCR Mix (Toyobo, Osaka, Japan) and threshold cycles (Ct) values were normalized to glyceraldehyde-3-phosphate dehydrogenase (GAPDH) expression. The expression of miR-203a-3p was quantified by a fast real-time PCR system (7900 HT, ABI, CA) and Ct values were normalized to U6 expression. The relative expression levels were determined according to the 2^−ΔΔ CT^ method. The sequences of primers used for PCR amplification are listed in Tables S3 and S4, see online supplementary material.

### *In situ* hybridization assay

Sections were deparaffinized, deproteinized and prehybridized at 42 °C for 2 h and then incubated with the DIG-labeled probe solution at 37 °C overnight. Thereafter, the sections were reacted with streptavidin-peroxidase and stained with DAB after stringent washing for 2 min. Then, the sections were counterstained for 5 min with 0.1% hematoxylin (Servicebio, Wuhan, China). miR-203a-3p expression levels were observed by a fully automatic pathological section scanner (KF-PRO-020, Ningbo, China).

### Dual luciferase reporter assay

To predict the potential binding sites between miR-203a-3p and the 3′UTR of PIK3CA, we searched the MiRDB database (http://mirdb.org). The potential binding sites of the PIK3CA-3′UTR were disrupted to create mutations. Thereafter, the wild type (PIK3CA-3′UTR-wt) and mutated type (PIK3CA-3’UTR-mut) were cloned and inserted into the GV272 luciferase reporter plasmid, which was cotransfected with miR-203a-3p mimics (Lv-miR-203a-3p) and mimics negative control (Lv-miR-NC) using X-tremegene HP (Roche, Basel, Switzerland). After 48 h of transfection, luminescence was detected using the dual luciferase reporter assay system (Promega, Fitchburg, WI, USA) according to the protocol. Data were normalized to Renilla luminescence and are presented relative to the Lv-miR-NC group.

### Isolation and culture of HHS fibroblasts

Primary HHS fibroblasts (HHSFs) were isolated from HHS tissues according to a standard protocol [16]. Thereafter, HHSFs were maintained in dulbecco’s modified eagle medium (DMEM) (Gibco, USA) containing 10% fetal bovine serum (Gibco, USA) at 37 °C with 5% CO_2_. Cells were passaged at 90% confluence and used in the experiments from passages 2 to 6.

### Lentiviral vector construction and generation of stable cell lines

Pre-miR-203a-3p lentivirus (Lv-miR-203a-3p), PIK3CA-overexpressing lentivirus (Lv-PIK3CA) and its negative control lentivirus (Lv-control), miR-203a-3p inhibitor lentivirus (Sh-miR-203a-3p) and its negative control lentivirus (Sh-control) were purchased from VectorBuilder (Guangdong, China). HHSFs were infected with lentivirus using polybrene (GeneChem, Shanghai, China) according to the manufacturer’s instructions. After 72 h of infection, 2 μg/ml puromycin was used to select stable clonal cell lines.

### Cell viability and cell cycle analysis

HHSFs were seeded in 96-well plates (3 × 10^3^ cells/well) and incubated for 24, 48, 72 and 96 h in triplicate. Cell viability was evaluated by cell counting kit-8 (CCK8; MedChemExpress, NJ, USA). During cell cycle analysis, HHSFs were harvested and fixed overnight in 75% ethanol. After washing twice with cold PBS, HHSFs were stained with PI/RNase staining buffer on ice and analyzed on a BD LSRFortessa analyzer (BD Biosciences, Franklin Lakes, USA).

### Scratch migration assay

HHSFs were plated in six-well plates and cultivated until 90% confluence. A scratch was created with a 200 μl sterilized pipette tip. Following the removal of debris, serum-free DMEM was added. We then obtained five randomly selected images from each well using a microscope at 0, 12, 24 and 48 h. The scratch areas at 0 h were set as 100% and the migration rates at various time points were compared.

### Collagen gel contraction assay

HHSFs were resuspended in rat tail type 1 collagen (Yeasen, Shanghai, China), and 1 ml of this suspension was plated in 12-well plates. The plates were then placed in a sterile incubator (37 °C) for 30 min to allow collagen gel polymerization. Thereafter, 1 ml of complete culture medium was added to each well. All the wells were photographed 12 and 24 h later, and ImageJ was used to measure the percent contraction.

### Western blot analysis

The collected HS samples and HHSFs were lysed with ice-cold radioimmunoprecipitation assay (RIPA) lysis buffer supplemented with a protease inhibitor cocktail and centrifuged for 10 min after sonication. After concentration detection, equal amounts of protein (20 μg) were separated by 8% SDS-PAGE and then electrotransferred onto polyvinylidene fluoride membranes, which were incubated overnight at 4 °C with primary antibodies after blocking with 5% BSA. Thereafter, the membranes were incubated with the corresponding horseradish peroxidase-conjugated secondary antibodies for 1 h at room temperature after removing excess primary antibodies. The signal was detected using a FluorChem E system (ProteinSimple, USA). Quantitative analysis was performed using ImageJ software. Antibody information is listed in Table S2, see online supplementary material.

### Transcriptome sequencing

Total RNA was extracted from HHSFs overexpressing miR-203a-3p and its negative control using TRIzol reagent (Invitrogen, CA, USA). The cDNA libraries were sequenced on the Illumina sequencing platform by Genedenovo Biotechnology (Guangzhou, China). The data presented in this study are representative of three independent sequencing experiments. DEGs were identified by the criteria of |logFC| ≥ 2 and adjusted *p* value < 0.05.

### Statistical analysis

Two-tailed Student’s *t* test was used for two-group comparisons, and one-way analysis of variance with postTukey’s multiple comparisons test and two-way analysis of variance with postSidak’s multiple comparisons test were used for comparisons of multiple groups. A Spearman correlation test was performed to determine the associations between PIK3CA and fibrosis extent in our included HHS samples. *P* < 0.05 was judged to be statistically significant. Three or more independent replicates were used for each experiment. All data in bar graphs are presented as the mean ± standard deviation.

## Results

### An RHS model analogous to HHS was successfully established

**Figure 1:**
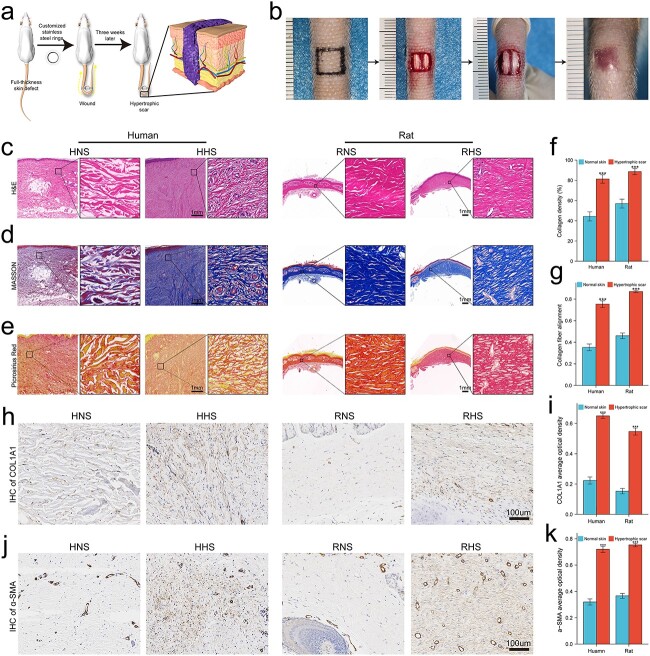
An RHS model similar to HHS was successfully prepared. (**a**) Schematic diagram of stretch-induced RHS model preparation. (**b**) Macroscopic photographs at each stage of RHS formation. (**c**) H&E staining shows the morphology of normal skin and hypertrophic scar tissues in human and rat; scale bar : 1 mm. (**d**) Masson staining shows the collagen deposition of normal skin and hypertrophic scar tissues in human and rat; scale bar: 1 mm. (**e**) Picrosirius staining shows the collagen distribution of normal skin and hypertrophic scar tissues in human and rat; scale bar : 1 mm. (**f**) Quantitative analysis shows collagen density in both HHS and RHS was increased. (**g**) Quantitative analysis shows that both HHS and RHS had more aligned collagen fibers than HNS and RNS. (**h**,**i**) Immunohistochemistry of COL1A1 and quantitative analysis; scale bar: 100 μm. (**j**,**k**) Immunohistochemistry of α-SMA and quantitative analysis; scale bar: 100 μm. ^*^^*^^*^p< 0.001. *HHS* Human hypertrophic scar, *HNS* human normal skin, *RHS* rat hypertrophic scar, *RNS* rat normal skin, *H&E* hematoxylin and eosin, *IHC* immunohistochemistry

Fibrotic repair, instead of regeneration, usually occurs in human cutaneous wounds. However, due to the presence of panniculus carnosus, contraction and re-epithelialization are the main mechanisms that drive murine dorsal wound healing, which makes scars atrophic and inconspicuous, hampering long-term studies of scar remodeling. Therefore, we drew on previously reported research on the novel stretch-induced scar model [17]. As shown in Figure 1a,b, after 3 weeks of tension induction, dark red RHS protruding from the skin surface was successfully prepared. In terms of histology, H&E staining showed that collagen fibers in RHS and HHS were more compact than those in the corresponding controls (Figure 1c). Masson staining showed that the collagen bundles in HNS and RNS were sparse, while the collagen fibers (dyed blue) in HHS and RHS were significantly increased, and the collagen fibers were arranged in an orderly fashion, indicating scar formation (Figure 1d). Moreover, picrosirius red staining revealed an increased distribution of collagen in HHS and RHS compared with normal skin controls (Figure 1e). The corresponding statistics of collagen density and fiber alignment are shown in Figure 1f,g. As hallmarks of fibrosis, the expression levels of collagen type I alpha 1 (COL1A1) and α-smooth muscle actin (α-SMA) were assessed by immunohistochemistry (IHC). Both COL1A1 and α-SMA protein levels were significantly higher in HHS and RHS than in normal skin controls (Figure 1h–k). Taken together, these results indicated that we successfully prepared the RHS model, which was analogous to HHS not only in appearance but also at the histological level.

### PIK3CA was overexpressed in both HHS and RHS tissues and closely related to fibrosis

**Figure 2:**
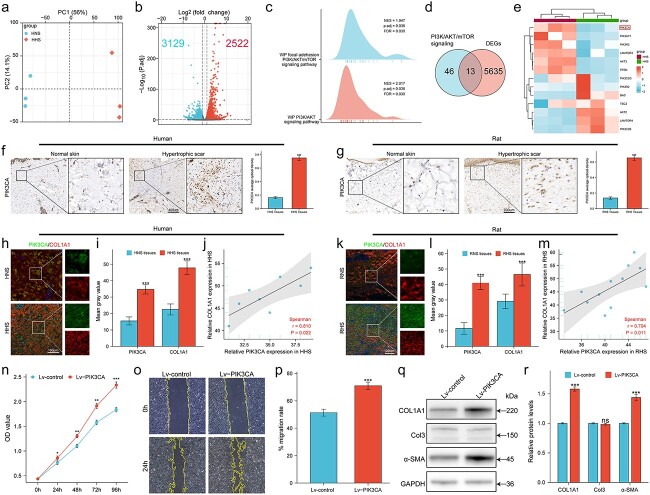
PIK3CA was overexpressed in hypertrophic scar and was closely related to fibrosis. (**a**) PCA was performed to visualize the separation of HHS tissues and adjacent HNS tissues (n = 3). (**b**) Volcano plot showing DEGs between HHS and HNS tissues. (**c**) GSEA analysis revealed a significant enrichment of PI3K/AKT/mTOR signaling. (**d**) Venn diagram showing the overlaps between DEGs and PI3K/AKT/mTOR signaling. (**e**) Heatmap showing expression profile of 13 candidate genes. (**f**) Immunohistochemistry analysis of HHS and HNS (n = 8); scale bar : 400 μm. (**g**) Immunohistochemistry analysis of RHS and RNS (n = 12); scale bar: 200 μm. (**h**–**j**) Immunofluorescence analysis of PIK3CA and COL1A1 in human tissues (n = 8); scale bar: 100 μm. (**k**–**m**) Immunofluorescence analysis of PIK3CA and COL1A1 in rat tissues (n = 12); scale bar : 100 μm. (**n**) Cell viability was assessed by CCK8 (n = 3). (**o**, **p**) The migration of HHSFs was quantified by scratch wound assay (n = 3). (**q**, **r**) Levels of COL1A1, Col3 and α-SMA were determined by WB analysis (n = 3). ns, no significance; ^*^^*^p < 0.01; ^*^^*^^*^p < 0.001. *PCA* principal component analysis, *DEGs* differentially expressed genes, *GSEA* gene set enrichment analysis, *CCK8* cell counting kit-8, *HHSFs* human hypertrophic scar fibroblasts, *WB* western blot, *HHS*, human hypertrophic scar, *HNS* human normal skin, *RHS* rat hypertrophic scar, *RNS* rat normal skin, *GAPDH* glyceraldehyde-3-phosphate dehydrogenase, *PIK3CA* phosphatidylinositol 3-kinase catalytic subunit alpha, *PI3K* phosphatidylinositol 3-kinase

To systematically investigate the mechanisms associated with HS pathogenesis, we retrieved the relevant datasets of HHS from the GEO database. PCA and hierarchical clustering showed that HNS and HHS had clear differences in clustering characteristics (Figure 2a and Figure S1a, see online supplementary material). A total of 5651 DEGs were identified in HHS compared to HNS, including 2522 upregulated and 3129 downregulated genes (Figure 2b). GO and KEGG pathway analysis uncovered that the most significant alterations in HHS were in extracellular matrix (ECM) and collagen synthesis (Figure S1b, see online supplementary material). Moreover, GSEA identified enhanced activity of multiple pathways in HHS, among which the PI3K/AKT/mTOR pathway was the most significant, indicating that inhibition of this pathway might be beneficial for the treatment of HS (Figure 2c). To identify the key genes regulating the pathway, we first identified 59 genes involved in the PI3K/AKT/mTOR pathway and subsequently performed Venn gathering analysis of these genes with DEGs (Figure 2d). The 59 PI3K/AKT/mTOR pathway-related genes are AKT1, AKT1S1, AKT2, AKT3, AKTIP, AMPK, BAD, DVL, ERK, FKBP, FOXO, GRB2, GSK3, IRS-1, LAMTOR1, LAMTOR2, LAMTOR3, LAMTOR4, LAMTOR5, LKB1, MEK, MTOR, NF1, PDK1, PIK3AP1, PIK3C2A, PIK3C2B, PIK3C2G, PIK3C3, PIK3CA, PIK3CB, PIK3CD, PIK3CG, PIK3IP1, PIK3R1, PIK3R2, PIK3R3, PIK3R4, PIK3R5, PIK3R6, PIKFYVE, PIP2, PIP3, PLD1, PP2A, PRAS40, PTEN, RAC, Raf, Ras, REDD1, REDD2, Rheb, RHO, RSK, SOS, TCTP, TSC1 and TSC2 [18,19]. A total of 13 DEGs were identified and the heatmap showed that PIK3CA was the most upregulated gene in HHS (Figure 2e).

To confirm the bioinformatics results, clinical specimens and animal specimens were analyzed. The results of IHC showed that PIK3CA was overexpressed in both HHS and RHS tissues (Figure 2f,g). These results were further supported by both real-time quantitative polymerase chain reaction (qRT-PCR) and western blot (WB) analyses (Figure S2, see online supplementary material). Moreover, the immunofluorescence (IF) results showed that PIK3CA and COL1A1 were both significantly upregulated in HS tissues, and these two proteins were positively correlated with each other in both HHS and RHS (Figure 2h–m). Furthermore, *in vitro* results showed that PIK3CA not only promoted HHSF proliferation, but also enhanced the motility and collagen synthesis (Figure 2n–r). Collectively, these results indicated that PIK3CA was overexpressed in both HHS and RHS tissues and positively related to fibrosis.

### miR-203a-3p could be a potential therapeutic molecule for HS by targeting PIK3CA

**Figure 3:**
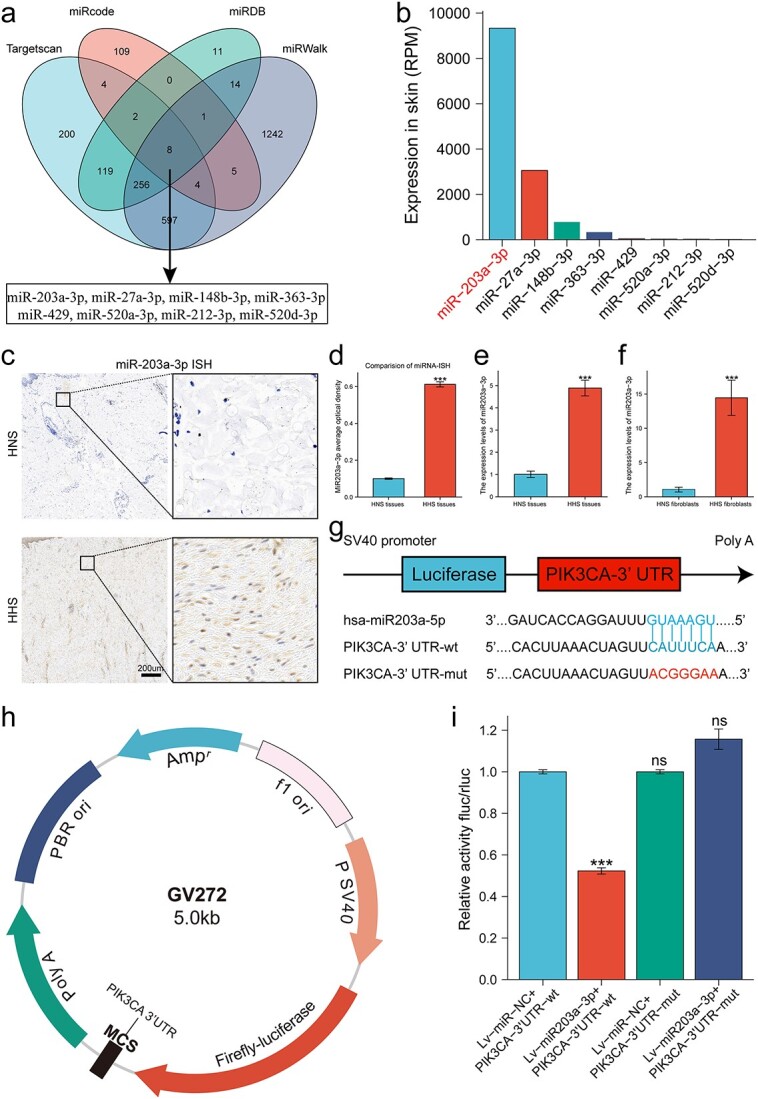
MiR-203a-3p could be a potential therapeutic molecule for HS by targeting PIK3CA. (**a**) Venn diagram of the candidate miRNAs targeting PIK3CA. (**b**) Bar graphs showing relative abundance of the candidate miRNAs in skin tissue. (**c**,**d**) Analysis of miR-203a-3p ISH (n = 8); scale bar : 200 μm. (**e**) qRT-PCR analysis of HHS and HNS tissues (n = 8). (**f**) qRT-PCR analysis of HHS and HNS fibroblasts (n = 8). (**g**) The binding sites between miR-203a-3p and PIK3CA-3′UTR. (**h**,**i**) Dual luciferase reporter assay (n = 3). ns, no significance; ^*^^*^^*^p < 0.001. *HS* hypertrophic scar, *ISH* in situ hybridization, *HHS* human hypertrophic scar, *HNS* human normal skin, *qRT-PCR* real-time quantitative polymerase chain reaction, *UTR* untranslated region

Considering the significant role of PIK3CA in HS, we explored miRNA targets of PIK3CA using several miRNA prediction databases. The intersection of four databases uncovered eight potential miRNAs targeting PIK3CA (Figure 3a). Among these miRNAs, miR-203a-3p showed the highest abundance in skin tissue based on the microRNA Tissue Expression Database (miTED) (Figure 3b). To validate the above bioinformatics results, an *in situ* hybridization (ISH) assay was implemented to determine the expression of miR-203a-3p in HHS and HNS. As expected, the expression of miR-203a-3p was noticeably elevated in HHS tissues (Figure 3c,d). Moreover, qRT-PCR results further supported the difference both in tissues and fibroblasts (Figure 3e,f). In addition, computational prediction suggested that the 3′UTR of PIK3CA contained miR-203a-3p binding sites (Figure 3g). A luciferase reporter assay was performed to confirm the target relationship. miR-203a-3p significantly reduced the luciferase activity of HEK293 cells transfected with the wild-type 3′UTR of PIK3CA, whereas the mutant 3′UTR of PIK3CA completely abolished this suppression, suggesting that miR-203a-3p directly bound to the 3′UTR of PIK3CA (Figure 3h,i). Therefore, we chose miR-203a-3p as a potential therapeutic molecule for HS.

### MiR-203a-3p exerted an antifibrotic effect on HHSFs

**Figure 4:**
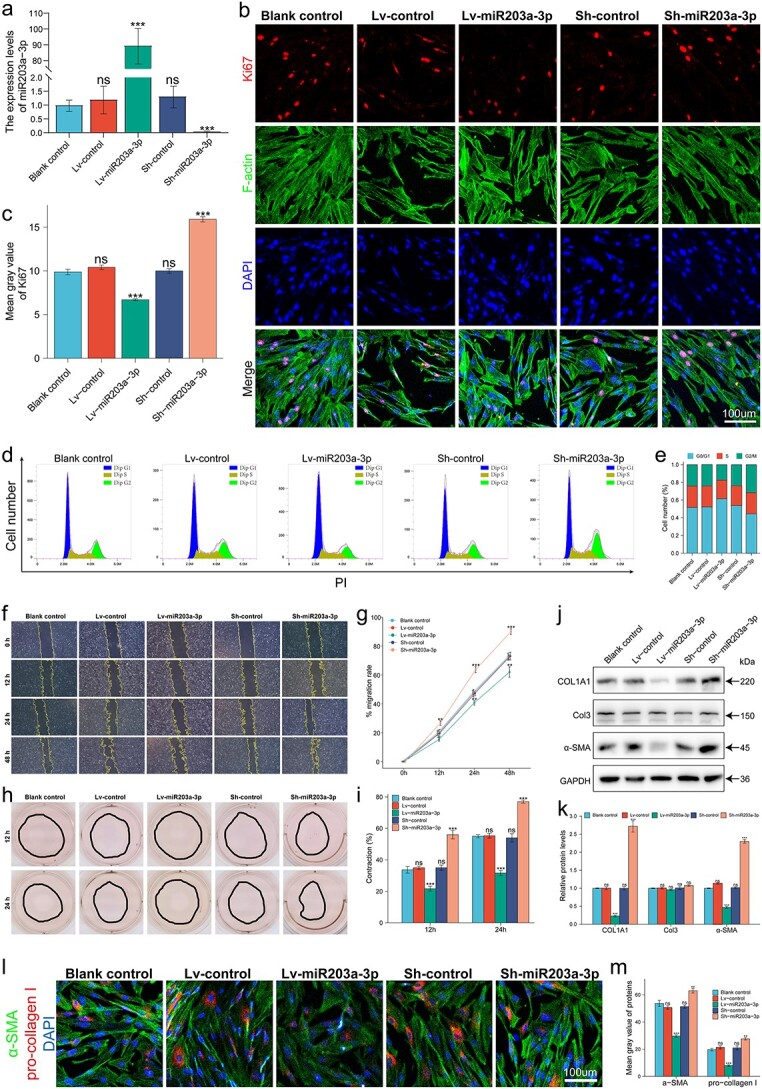
MiR-203a-3p exerted an anti-fibrotic effect on HHSFs. HHSFs were infected with miR-203a-3p overexpression lentivirus (Lv-miR-203a-3p), miR-203a-3p-knockdown lentivirus (Sh-miR-203a-3p) or corresponding control lentivirus (Lv-control or Sh-control). (**a**) Validation of the efficiency of lentiviral infection through qRT-PCR. (**b**,**c**) Analysis of Ki67 immunofluorescence (n = 3); scale bar : 100 μm; (**d**,**e**) Cell cycle was detected by flow cytometry. (**f**,**g**) Migration of HHSFs quantified by scratch wound assay (n = 3). (**h**, **i**) Contractility of HHSFs quantified by collagen contraction assay (n = 3). (**j**,**k**) Levels of COL1A1, Col3 and α-SMA quantified by WB analysis (n = 3). (**l**,**m**) Levels of α-SMA and pro-collagen I determined by immunofluorescence (n = 3); scale bar: 100 μm. ns, no significance; ^*^^*^p < 0.01; ^*^^*^^*^p < 0.001. *HHSFs* human hypertrophic scar fibroblasts, *WB* western blot, *GAPDH *glyceraldehyde-3-phosphate dehydrogenase, *α-SMA* α-smooth muscle actin

To examine the effect of miR-203a-3p, HHSFs with overexpression or inhibition of miR-203a-3p and the corresponding controls were constructed by lentiviral vectors (Figure 4a). In terms of proliferation, Ki67 staining showed that HHSFs overexpressing miR-203a-3p had a fluorescence intensity significantly lower than that in the control group, while a reverse trend was observed in HHSFs inhibiting miR-203a-3p (Figure 4b,c). In addition, flow cytometry analysis also showed that miR-203a-3p inhibited HHSF proliferation by prolonging the G0/G1 phase and shortening the S phase, while HHSFs inhibiting miR-203a-3p presented an opposite tendency (Figure 4d,e). Moreover, according to the live/dead staining results and CCK8 assays, HHSFs overexpressing miR-203a-3p showed a pronounced decrease in cell viability and cell proliferative activity, whereas an opposite trend was observed in HHSFs with miR-203a-3p inhibition (Figure S3, see online supplementary material). In terms of migration, the wound scratch assay showed that the mobility of HHSFs was significantly inhibited in response to miR-203a-3p stimulation, while inhibition of miR-203a-3p exerted the opposite effect (Figure 4f,g). In terms of collagen contraction, the results showed that miR-203a-3p reduced the collagen contractility of HHSFs, while collagen contractility was significantly increased by miR-203a-3p inhibition (Figure 4h,i). In terms of ECM synthesis, the WB results showed that miR-203a-3p markedly reduced the expression of COL1A1 and α-SMA, whereas inhibition of miR-203a-3p increased COL1A1 and α-SMA levels (Figure 4j,k). In terms of transdifferentiation, the expression of α-SMA and pro-collagen I was inhibited simultaneously when miR-203a-3p was overexpressed, while the opposite effect was observed when miR-203a-3p was knocked down, indicating that miR-203a-3p inhibited the transdifferentiation of HHSFs into myofibroblasts (Figure 4l,m). Together, these results demonstrated that miR-203a-3p significantly suppressed fibrosis progression by inhibiting HHSF proliferation, migration, collagen synthesis, contractility and transdifferentiation.

### MiR-203a-3p inhibited PIK3CA and the PI3K/AKT/mTOR pathway in HHSFs

**Figure 5:**
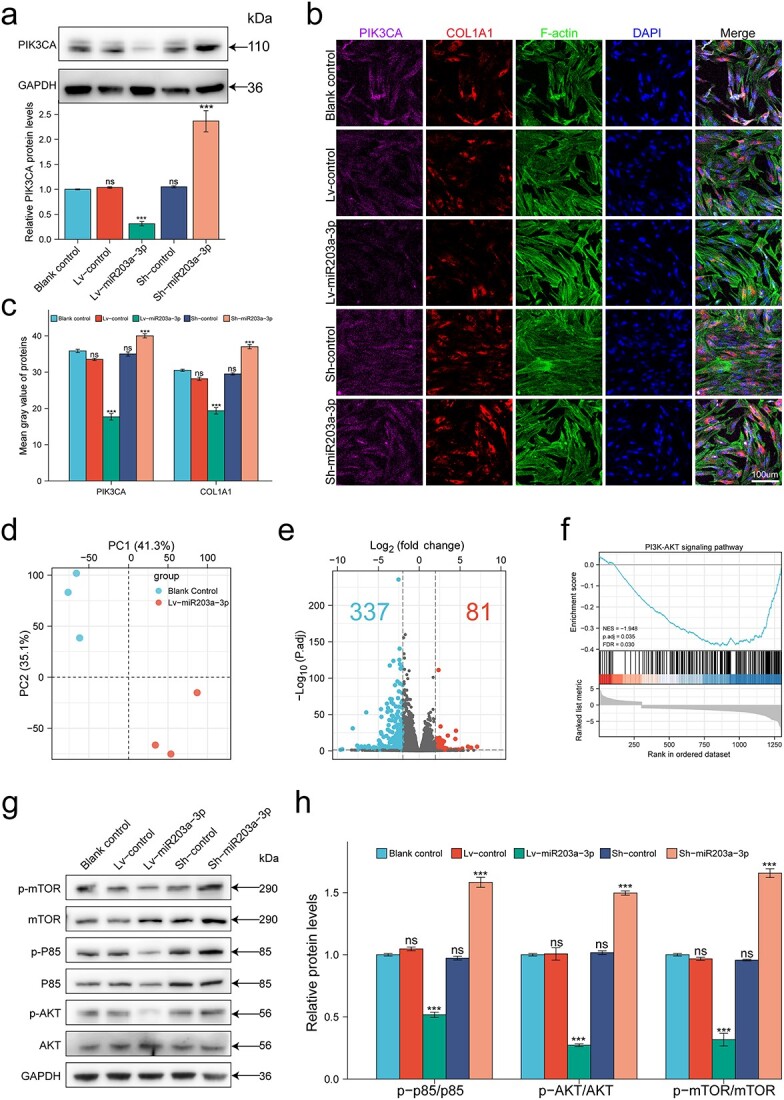
MiR-203a-3p inhibited PIK3CA and the PI3K/AKT/mTOR pathway in HHSFs. (**a**–**c**) Transcriptomic analyses were conducted with mRNAs from HHSFs overexpressing miR-203a-3p and corresponding controls (n = 3). (**a**) WB analysis confirmed the expression of PIK3CA was negatively correlated with miR-203a-3p levels. (**b**) Levels of PIK3CA and COL1A1 were determined by immunofluorescence (n = 3); scale bar = 100 μm. (**c**) Statistical analysis of PIK3CA and COL1A1, n = 3. (**d**) Principal component analysis. (**e**) Volcano plots showing DEGs. (**f**) GSEA revealed that PI3K/AKT pathway was significantly down-regulated upon miR-203a-3p overexpression. (**g**) Levels of p-mTOR, mTOR, p-P85, P85, p-AKT and AKT were determined by WB. (**h**) Statistical analysis of mTOR, P85 and AKT phosphorylation, n = 3. ns, no significance; ^*^^*^^*^p < 0.001. HHSFs human hypertrophic scar fibroblasts, *WB* western blot, *DEGs* differentially expressed genes, *GSEA* gene set enrichment analysis, *GAPDH* glyceraldehyde-3-phosphate dehydrogenase, *PIK3CA* phosphatidylinositol 3-kinase catalytic subunit alpha

To verify the targeting relationship between miR-203a-3p and PIK3CA, we quantified the expression levels of PIK3CA by WB. The results showed that the expression of PIK3CA was considerably reduced after overexpressing miR-203a-3p in HHSFs, while inhibition of miR-203a-3p increased PIK3CA protein levels (Figure 5a). The IF results showed that PIK3CA and COL1A1 were both significantly decreased in HHSFs overexpressing miR-203a-3p, whereas HHSFs with inhibition of miR-203a-3p displayed elevated levels of PIK3CA and COL1A1 (Figure 5b,c). To further explore the potential mechanisms underlying the antifibrotic effect of miR-203a-3p on HHSFs, RNA-sequencing was performed, and the results showed that HHSFs overexpressing miR-203a-3p and their negative control had clear differences in clustering characteristics (Figure 5d and Figure S4a, see online supplementary material). Compared with those in the controls, a total of 418 DEGs were identified in HHSFs overexpressing miR-203a-3p, including 81 upregulated and 337 downregulated genes (Figure 5e). Regarding signaling pathways, both KEGG analysis and GSEA revealed that the PI3K/AKT/mTOR pathway was one of the most significantly downregulated pathways identified upon miR-203a-3p overexpression (Figure 5f, Figure S4b, see online supplementary material). The WB results further confirmed that phosphorylation of the PI3K/AKT/mTOR pathway was markedly reduced in response to miR-203a-3p overexpression, while hyperactivation of this pathway was detected when miR-203a-3p was inhibited (Figure 5g,h). Collectively, these results demonstrated that in addition to the inhibitory effect on fibrosis, miR-203a-3p also inhibited the expression of PIK3CA and the activation of the PI3K/AKT/mTOR pathway.

### miR-203a-3p mitigated fibrosis by inactivating the PI3K/AKT/mTOR pathway by targeting PIK3CA

**Figure 6:**
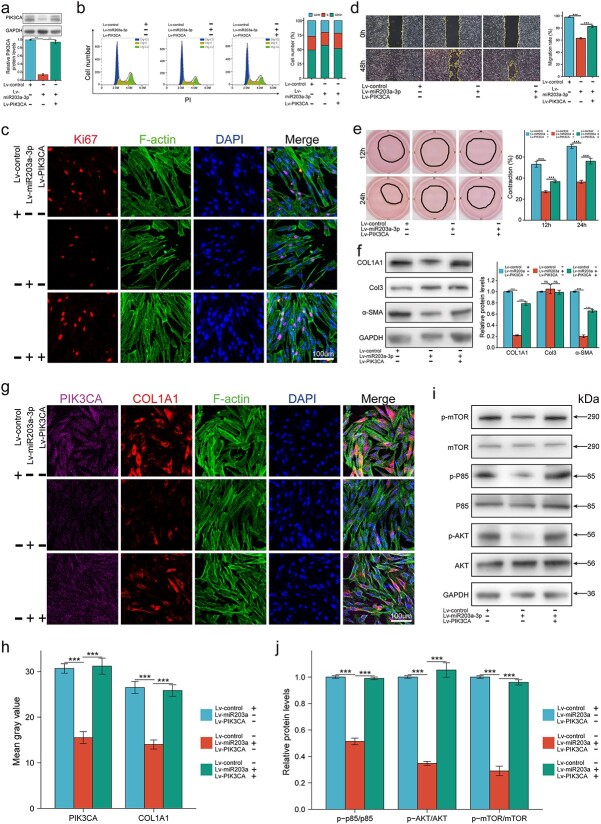
MiR-203a-3p mitigated fibrosis by inactivating the PI3K/AKT/mTOR pathway via targeting PIK3CA. HHSFs were infected with miR-203a-3p overexpression lentivirus (Lv-miR203a), or co-infected with PIK3CA overexpression lentivirus (Lv-PIK3CA) and Lv-miR203a. (**a**) Expression of PIK3CA was determined by WB (n = 3). (**b**) Cell cycle was detected by flow cytometry. (**c**) Levels of Ki67 were determined by immunofluorescence (n = 3); scale bar: 100 μm. (**d**) Migration of HHSFs was quantified by scratch wound assay (n = 3). (**e**) Contractility of HHSFs was quantified by collagen contraction assay (n = 3). (**f**) Levels of COL1A1, Col3, and α-SMA were determined by WB (n = 3). (**g**,**h**) Levels of PIK3CA and COL1A1 were determined by immunofluorescence (n = 3); scale bar:100 μm. (**i**,**j**) Phosphorylation levels of PI3K/AKT/mTOR pathway were determined by WB (n = 3). ^*^^*^^*^p < 0.001. *WB* western blot, *HHSFs* human hypertrophic scar fibroblasts, *PIK3CA* phosphatidylinositol 3-kinase catalytic subunit alpha, *GAPDH* glyceraldehyde-3-phosphate dehydrogenase,* α-SMA* α-smooth muscle actin

To investigate whether miR-203a-3p mitigated fibrosis by directly targeting PIK3CA, we co-overexpressed PIK3CA in HHSFs stably overexpressing miR-203a-3p (Figure 6a). In terms of proliferation, flow cytometry analysis revealed that miR-203a-3p hampered HHSFs proliferation by increasing the proportion of cells in G0/G1 phase, while co-overexpressing PIK3CA partially restored the proliferative capacity (Figure 6b). In addition, immunofluorescence staining of Ki67 showed that HHSFs overexpressing miR-203a-3p had lower expression of Ki67 than those in the control group, which was reversed by PIK3CA co-overexpression (Figure 6c). In terms of migration, miR-203a-3p inhibited HHSFs motility, while co-overexpressing PIK3CA partially rescued the inhibitory effect (Figure 6d). In terms of collagen contraction, contractility was significantly impaired when miR-203a-3p was overexpressed, while the inhibitory effect was diminished by PIK3CA co-overexpression (Figure 6e). In terms of ECM synthesis, the WB results showed that miR-203a-3p significantly reduced the expression of COL1A1 and α-SMA, whereas PIK3CA co-overexpression partially rescued the levels of these two proteins (Figure 6f). In terms of transdifferentiation, the expression of α-SMA and pro-collagen I was inhibited by miR-203a-3p overexpression, while PIK3CA overexpression partially recovered the inhibitory effect of miR-203a-3p overexpression (Figure S5, see online supplementary material). In addition, the IF results demonstrated that miR-203a-3p resulted in a significant reduction in PIK3CA and COL1A1, while co-overexpression of PIK3CA partially offset the inhibitory effect, indicating that the miR-203a-3p/PIK3CA axis played a crucial role in HHSFs fibrosis (Figure 6g,h). In terms of mechanisms, WB results showed that miR-203a-3p inhibited the phosphorylation of PI3K/AKT/mTOR signaling, while co-overexpression of PIK3CA rescued the phosphorylation-mediated activation of this pathway (Figure 6I,j). Taken together, these results showed that miR-203a-3p mitigated fibrosis by inactivating the PI3K/AKT/mTOR pathway by targeting PIK3CA.

### miR-203a-3p attenuated excessive scarring *in vivo* by targeting PIK3CA

**Figure 7:**
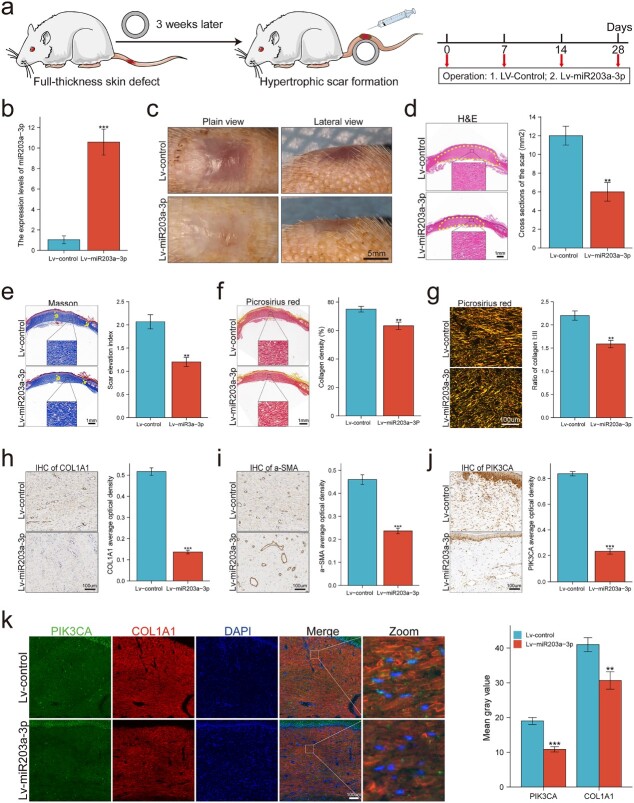
MiR-203a-3p attenuated excessive scarring *in vivo* through targeting PIK3CA. Lv-control or Lv-miR203a-3p were administrated subcutaneously once weekly after scar formation. (**a**) Schematic representation of animal model preparation and administration in the scar. (**b**) Expression level of miR-203a-3p between the two groups. (**c**) Morphology of RHS; scale bar : 5 mm. (**d**) H&E staining and analysis of scar cross sections (n = 12); scale bar = 1 mm. (**e**) Masson staining and analysis of scar elevation index (n = 12); scale bar : 1 mm. (**f**) Picrosirius staining and analysis of collagen density (n = 12); scale bar : 1 mm. (**g**) Picrosirius birefringence and analysis of collagen ratio (n = 12); scale bar : 100 μm. (**h**) Immunohistochemistry and analysis of (**i**) COL1A1 (n = 12); scale bar = 100 μm, (**j**) α-SMA (n = 12); scale bar : 100 μm and (**k**) PIK3CA (n = 12); scale bar : 100 μm. (**k**) Representative images of immunofluorescence and quantitative analysis showed both PIK3CA and COL1A1 were inhibited by miR-203a-3p *in vivo* (n = 12); scale bar : 100 μm. ^*^^*^p < 0.01; ^*^^*^^*^p < 0.001. *H&E* Hematoxylin and eosin, *PIK3CA* phosphatidylinositol 3-kinase catalytic subunit alpha, *α-SMA* α-smooth muscle actin, *IHC* immunohistochemistry

**Figure 8:**
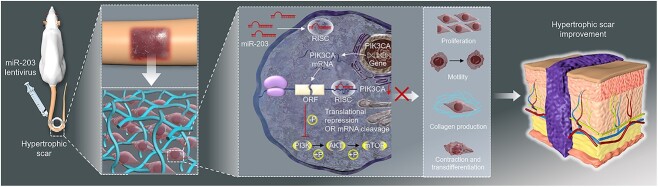
miR-203a-3p ameliorated HS formation through PIK3CA suppression and subsequent inactivation of PI3K/Akt/mTOR pathway. *PIK3CA* phosphatidylinositol 3-kinase catalytic subunit alpha, *RISC* RNA-induced silencing complex, *ORF* open reading frame, *HS* hypertrophic scar

Considering the antifibrotic effects of miR-203a-3p *in vitro*, we established an RHS model to investigate the therapeutic potential of miR-203a-3p *in vivo*. As shown in Figure 7a, 3 weeks after the operation, the skin defect was replaced by HS. Thereafter, in the next 4 weeks, control lentivirus (Lv-control) or miR-203a-3p-overexpressing lentivirus (Lv-miR-203a-3p) was subcutaneously administered once weekly according to the different groups. The expression level of miR-203a-3p in the Lv-miR-203a-3p group was more than 10-fold higher than that in the Lv-control group (Figure 7b). Macroscopic photographs are shown to display the scar changes: HSs treated with Lv-miR-203a-3p became remarkably thinner and softer and had a better clinical appearance than HSs treated with Lv-control (Figure 7c). Histological analysis further illustrated that the scar volume and the scar elevation index were significantly decreased in miR-203a-3p-treated scars compared with those in the controls (Figure 7d,e). Moreover, RHS tissues were stained with picrosirius red to evaluate collagen deposition. The results showed that miR-203a-3p significantly reduced collagen density in the scar (Figure 7f). In addition, picrosirius red birefringence was captured to determine the proportion of different collagen types. As shown in Figure 7g, type I collagen deposition and scar index (the ratio of collagen I/III) were markedly decreased in miR-203a-3p-treated scars. More importantly, the collagen fibers in the treated group were intermixed in a basket weave orientation, indicating normal healing, whereas the collagen fibers in the control group were oriented in parallel, indicating fibrosis. As biomarkers of fibrosis, α-SMA and COL1A1 expression levels in RHS were markedly downregulated after overexpression of miR-203a-3p (Figure 7h,i). In terms of the mechanism, IHC staining showed that miR-203a-3p treatment potently reduced PIK3CA expression in RHS tissues (Figure 7j). Consistently, either PIK3CA or COL1A1 expression was significantly decreased in miR-203a-3p-treated scars, and COL1A1 levels exhibited a significant positive correlation with PIK3CA, much as we observed *in vitro* (Figure 7k). Collectively, these findings illustrated that miR-203a-3p was also efficacious in mitigating HS formation *in vivo*, at least in part, by targeting PIK3CA (Figure 8).

## Discussion

In the present study, we demonstrated for the first time that miR-203a-3p reduced HHSF fibrosis *in vitro* and improved HS appearance *in vivo* by inhibiting the PI3K/AKT/mTOR pathway through directly targeting PIK3CA. Specifically, we showed that first, hyperactivation of the PI3K/AKT/mTOR signaling pathway and overexpression of PIK3CA were the potential etiological factors of HS, verifying that PIK3CA could enhance HHSF fibrotic function. Second, miR-203a-3p was confirmed to be a target miRNA of PIK3CA by applying bioinformatics algorithms. Third, miR-203a-3p attenuated HHSF proliferation, migration, collagen contraction, ECM synthesis and transdifferentiation *in vitro* and mitigated excessive scarring *in vivo* by suppressing PIK3CA expression. Fourth, miR-203a-3p inhibited the PI3K/AKT/mTOR signaling pathway by directly targeting PIK3CA. These findings suggested that miR-203a-3p ameliorated HS formation through PIK3CA suppression and subsequent inactivation of the PI3K/Akt/mTOR pathway.

The PIK3CA gene encodes the p110α protein, a catalytic subunit of PI3K, which is a vital component of the PI3K/Akt/mTOR signaling pathway [20]. PIK3CA plays a crucial role in multiple biological processes, including cell proliferation, migration and survival. Dysregulation of PIK3CA has been reported to be associated with a wide range of diseases [21]. Specifically, mutations in PIK3CA resulted in poor outcomes in metastatic breast cancer patients and resistance to chemotherapy [4]; the loss of PIK3CA suppressed cancer cell growth and migration in malignancies [8,22]. In addition to tumorigenesis, aberrant PIK3CA was also found in benign overgrowth syndromes: it was first identified as a critical gene associated with excessive scarring in a study that was conducted among patients with PIK3CA-related overgrowth syndromes [11]. However, since then no further research has been conducted to evaluate the effect of PIK3CA on HS. Here, we first determined that PIK3CA was overexpressed and positively correlated with fibrosis extent in both HHS and RHS tissues.

Investigation of HS has long been hindered by the lack of ideal animal models. Due to the presence of panniculus carnosus, the scar in the traditional rodent model becomes atrophic and inconspicuous soon after wound healing [23]. Therefore, this study successfully prepared fibroproliferative RHS similar to HHS by referring to the novel stretch-induced rat tail scar model [17]. It has been widely acknowledged that biomechanics play a crucial role in fibroproliferative disorders. Fibroblasts possess the ability to perceive alterations in their physical surroundings and subsequently convert extracellular mechanical signals into intracellular biochemical reactions and gene expression regulation. Specifically, the process consists of cellular mechanosensing, mechanotransduction and mechanoeffecting, which have been confirmed to be involved in molecules and signaling pathways, including Piezo, leucine rich alpha-2-glycoprotein 1 (LRG-1), the Hippo signaling pathway, the Wnt/β-catenin signaling pathway, the interleukin 6 (IL-6)/signal transducer and activator of transcription 3 (STAT3) signaling pathway and the FAK signaling pathway [3,24–26]. Our study demonstrated that hyperactivation of the PI3K/AKT/mTOR pathway and overexpression of PIK3CA play an important role in stretch-induced hypertrophic scarring, which is an important complement to the mechano-regulatory axis in fibrosis.

Based on the above research, we proved that PIK3CA played a pivotal role in HS formation. Therefore, seeking a PIK3CA-targeting inhibitor may provide potential therapeutic benefit for HS prevention and therapy. Despite their short length, miRNAs are involved in almost all biological processes and have been found to be critical in HS formation: miR-31-5p was confirmed to promote fibroblasts proliferation, invasion, and ECM deposition; conversely, fibroblast proliferation and collagen deposition were inhibited by miR-519d in HS [27,28]. Moreover, miRNAs have proven to be effective as therapeutics in various diseases, including HS [28, 29]. For example, miR-296 was down-regulated and its mimics proved to be potent therapeutics to prevent excessive scar in rabbit HS [30]. Hence, we aimed to identify one miRNA directly targeting PIK3CA that might potentially attenuate the progression of HS. The intersection of multiple prediction websites indicated that miR-203a-3p was the most suitable candidate to inhibit PIK3CA.

Previous studies have demonstrated that miR-203 plays a pivotal role in various fibrotic diseases, including cardiac fibrosis, pulmonary fibrosis and bladder fibrosis [13,14,31]. Here, we found that HS tissues and fibroblasts exhibited high expression of miR-203a-3p. It is widely known that fibroblasts are crucial for HS formation [32]. A previous study emphasized that miR-203 had a therapeutic effect on keloids through suppression of fibroblast survival pathways [33]. In this study, we demonstrated that miR-203a-3p suppressed HHSF proliferation in a PIK3CA-mediated manner. In addition to proliferation, fibroblasts migration into the wound clot is considered to be a significant contributor to HS formation [34]. Previous studies have shown that miR-203 inhibits the migration and invasive capacity of tumor cells [35,36]. Our *in vitro* scratch assay also suggested that miR-203a-3p markedly suppressed HHSF mobility. Moreover, there is substantial evidence that excessive ECM deposition leads to HS formation [37]. Several studies have shown that miR-203 plays a pivotal role in regulating ECM deposition [38,39]. We observed that the COL1A1 content in HHSFs was reduced by overexpressing miR-203a-3p, indicating that miR-203a-3p plays an essential role in suppressing excessive ECM deposition. Furthermore, transdifferentiation of fibroblasts into myofibroblasts is another key factor directly related to HS formation, while α-SMA and pro-collagen I are presumed to be a characteristic markers of myofibroblasts [40]. In this study, we found that miR-203a-3p diminished the expression of α-SMA and pro-collagen I simultaneously and inhibited collagen contractility, indicating that miR-203a-3p has the potential to inhibit myofibroblasts conversion. Taken together, our findings illustrated that miR-203a-3p suppressed the proliferation, migration, collagen synthesis and contractility, as well as the transdifferentiation of HHSFs.

For the mechanisms underlying miR-203a-3p-mediated HS mitigation, we employed a bioinformatics approach to screen possible signaling pathways. The data revealed that the expression of PIK3CA and the PI3K/AKT/mTOR pathway were significantly down-regulated after miR-203a-3p overexpression, which is in concordance with previous findings [10,41]. The PI3K/AKT/mTOR pathway has been identified in numerous studies as a fibrosis signaling pathway: it was confirmed to promote myofibroblasts transformation and augment collagen strength in cardiac fibrosis, and activation of this pathway could stimulate collagen synthesis in lung fibroblasts through aerobic glycolysis [42,43]. In addition, the PI3K/AKT/mTOR pathway also plays an important role in keloids and liver fibrosis [44,45]. Consistently, our rescue experiments clarified that the anti-fibrosis and inactivation of the PI3K/AKT/mTOR pathway, initiated by miR-203a-3p, were dependent on PIK3CA. Taken together, our findings illustrated that miR-203a-3p inhibited HHSF fibrosis by directly suppressing PIK3CA, followed by inactivation of the PI3K/AKT/mTOR pathway.

To explore the *in vivo* effects of miR-203a-3p, we chose the novel stretch-induced scar model. Traditional rodent models of HS are flawed due to rapid wound contraction and unapparent scarring after healing, which is different from HHS [46]. In our study, the RHS model exhibited similar appearance and pathological features to HHS, further corroborating the superiority of this novel HS model [17]. After regional injection of miR-203a-3p, the scar appearance was noticeably improved, while the cross-sectional area, scar elevation index, collagen density and scar index were significantly decreased, indicating that miR-203a-3p improved the morphology and histological phenotypes of scars *in vivo* [47]. Previous studies have shown that α-SMA is highly expressed in fibrotic disease-derived myofibroblasts [48,49]. Our IHC results also showed a significant reduction in α-SMA in miR-203a-3p-treated scars, suggesting that miR-203a-3p inhibited fibroblasts transdifferentiation *in vivo*. Moreover, the expression of both PIK3CA and COL1A1 was downregulated after miR-203a-3p treatment, which was in line with our *in vitro* observations.

Whilst these findings suggest promising therapeutic potential for miR-203a-3p to ameliorate scarring, it is prudent to consider the potential limitations. First, the clinical sample size is relatively small, and second, no miRNA-based medication *in vivo* is currently available for clinical use, although some are now currently being trialed for HS (NCT03601052).

## Conclusions

In summary, our study confirmed that overexpression of PIK3CA and hyperactivation of PI3K/AKT/mTOR signaling were involved in HS formation. We identified miR-203a-3p as a specific inhibitor of PIK3CA using a bioinformatics approach, and miR-203a-3p reduced HS formation by inhibiting HHSFs proliferation, migration, collagen contraction, ECM deposition and fibroblast transdifferentiation, accompanied by the inactivation of the PI3K/AKT/mTOR pathway. Our results shed light on new potential therapeutic targets in HS.

## Supplementary Material

Figure_S1_tkad048

Figure_S2_tkad048

Figure_S3_tkad048

Figure_S4_tkad048

Figure_S5_tkad048

Table_S1_tkad048

Table_S2_tkad048

Table_S3_tkad048

Table_S4_tkad048

## Data Availability

The data used to support the findings of this study are available from the corresponding author upon request. The raw sequencing data from this study have been deposited in the GEO database with the primary accession code GSE236983 and in BioProject with the primary accession code PRJNA993252.
