# Supplementary material for: Inhibition of phosphatidylinositol 3-kinase catalytic subunit alpha by miR-203a-3p reduces hypertrophic scar formation via phosphatidylinositol 3-kinase/AKT/mTOR signaling pathway
Source: Burns Trauma. 2024 Jan 2;12:tkad048. doi: 10.1093/burnst/tkad048 (PMC10762504; doi:10.1093/burnst/tkad048)
Supplement: Figure_S1_tkad048 [file figure_s1_tkad048.docx]

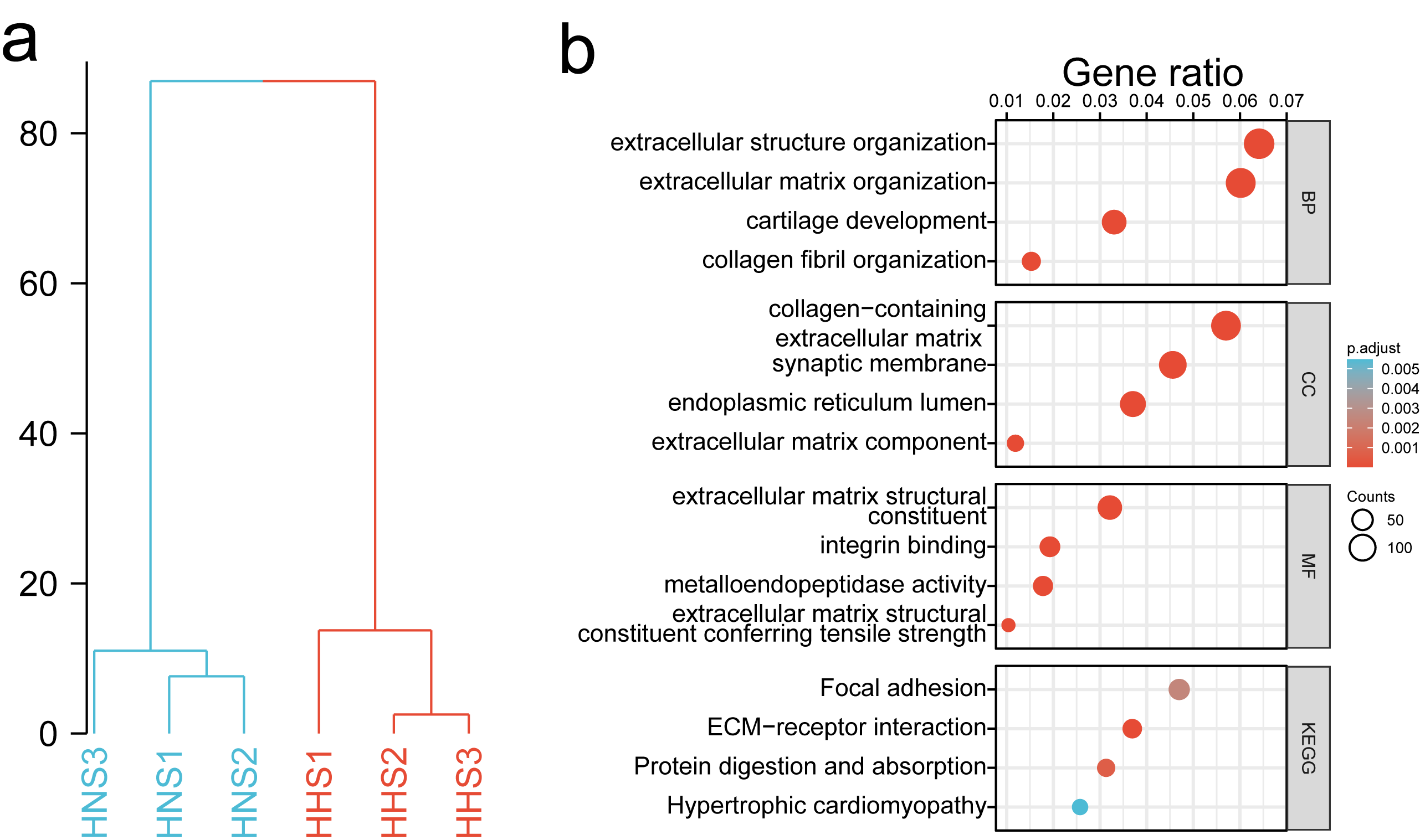


**Figure S1. Transcriptomic analysis between HHS and HNS.** (**a**) Cluster dendrogram confirmed that HHS and HNS had different enrichment signatures. (**b**) GO and KEGG analysis showed that the most significant alterations in HHS were ECM and collagen synthesis. *HHS* human hypertrophic scar, *HNS* human normal skin, *GO* gene ontology, *KEGG* Kyoto Encyclopedia of Genes and Genomes, *ECM* extracellular matrix
