# Supplementary material for: Inhibition of phosphatidylinositol 3-kinase catalytic subunit alpha by miR-203a-3p reduces hypertrophic scar formation via phosphatidylinositol 3-kinase/AKT/mTOR signaling pathway
Source: Burns Trauma. 2024 Jan 2;12:tkad048. doi: 10.1093/burnst/tkad048 (PMC10762504; doi:10.1093/burnst/tkad048)
Supplement: Figure_S2_tkad048 [file figure_s2_tkad048.docx]

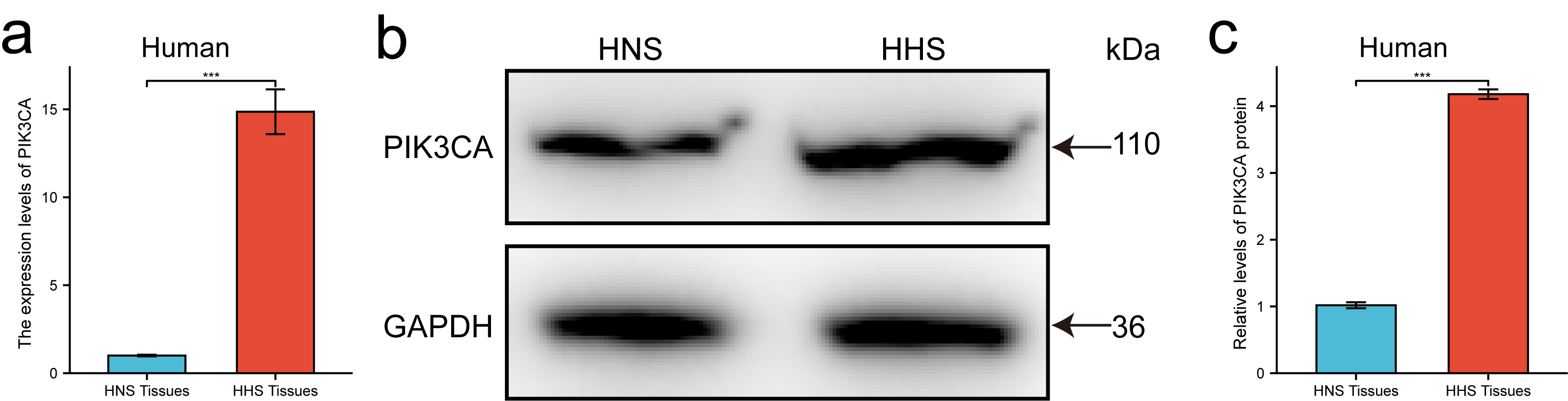


**Figure S2. The expression of PIK3CA in HHS was significantly higher than that in HNS.** (**a**) QRT-PCR results suggested that PIK3CA mRNA content in HHS was approximately 14 times higher than in HNS. (**b-c**) WB images and quantitative analysis showed that PIK3CA was overexpressed in HHS than in HNS. *** *p* < 0.001. *HHS* human hypertrophic scar, *HNS* human normal skin, *QRT-PCR* real-time quantitative polymerase chain reaction, *WB* western blot
