# Supplementary material for: Inhibition of phosphatidylinositol 3-kinase catalytic subunit alpha by miR-203a-3p reduces hypertrophic scar formation via phosphatidylinositol 3-kinase/AKT/mTOR signaling pathway
Source: Burns Trauma. 2024 Jan 2;12:tkad048. doi: 10.1093/burnst/tkad048 (PMC10762504; doi:10.1093/burnst/tkad048)
Supplement: Figure_S3_tkad048 [file figure_s3_tkad048.docx]

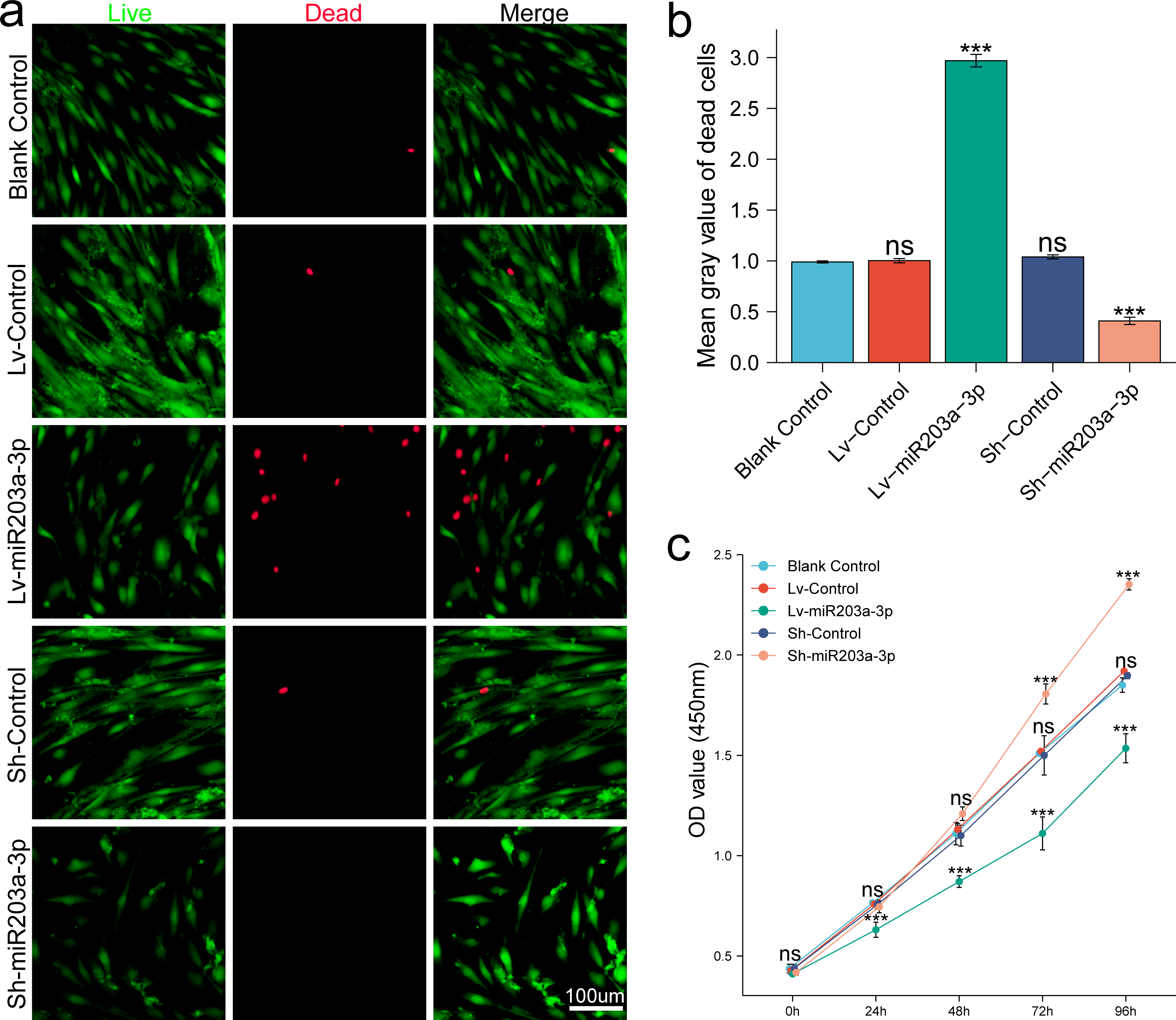


**Figure S3. MiR-203a-3p affected HHSFs viability.** (**a-b**) Representative images of live/dead staining and quantitative analysis of HHSFs with different miR-203a-3p levels, Scale bar: 100μm. (**c**) CCK8 assay showed the viability of HHSFs overexpressing miR-203a-3p was lower than that of the control group, while HHSFs inhibiting miR-203a-3p had the opposite trend. ns, no significance; ****p* < 0.001. *HHSFs* human hypertrophic scar fibroblasts, *CCK8* cell counting kit-8
