# Supplementary material for: Inhibition of phosphatidylinositol 3-kinase catalytic subunit alpha by miR-203a-3p reduces hypertrophic scar formation via phosphatidylinositol 3-kinase/AKT/mTOR signaling pathway
Source: Burns Trauma. 2024 Jan 2;12:tkad048. doi: 10.1093/burnst/tkad048 (PMC10762504; doi:10.1093/burnst/tkad048)
Supplement: Figure_S4_tkad048 [file figure_s4_tkad048.docx]

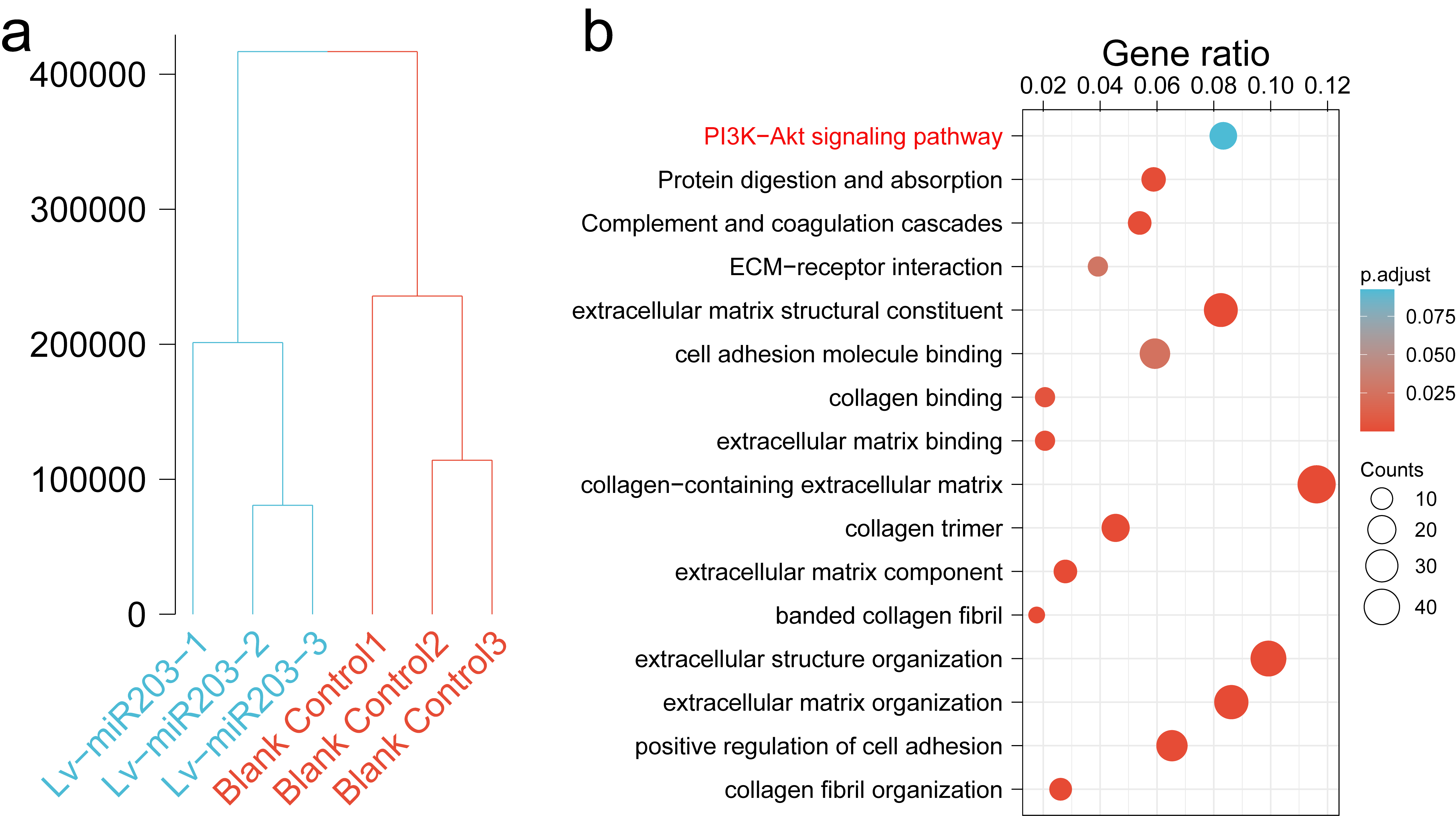


**Figure S4. Transcriptomic analysis between** **HHSFs overexpressing miR-203a-3p and controls.** (**a**) Cluster dendrogram confirmed that HHSFs overexpressing miR-203a-3p and controls had different enrichment signatures. (**b**) GO and KEGG analysis identified PI3K/AKT signaling pathway was the most significantly altered biological process. *HHSFs* human hypertrophic scar fibroblasts, *GO* gene ontology, *KEGG* Kyoto Encyclopedia of Genes and Genomes
