# Supplementary material for: Inhibition of phosphatidylinositol 3-kinase catalytic subunit alpha by miR-203a-3p reduces hypertrophic scar formation via phosphatidylinositol 3-kinase/AKT/mTOR signaling pathway
Source: Burns Trauma. 2024 Jan 2;12:tkad048. doi: 10.1093/burnst/tkad048 (PMC10762504; doi:10.1093/burnst/tkad048)
Supplement: Figure_S5_tkad048 [file figure_s5_tkad048.docx]

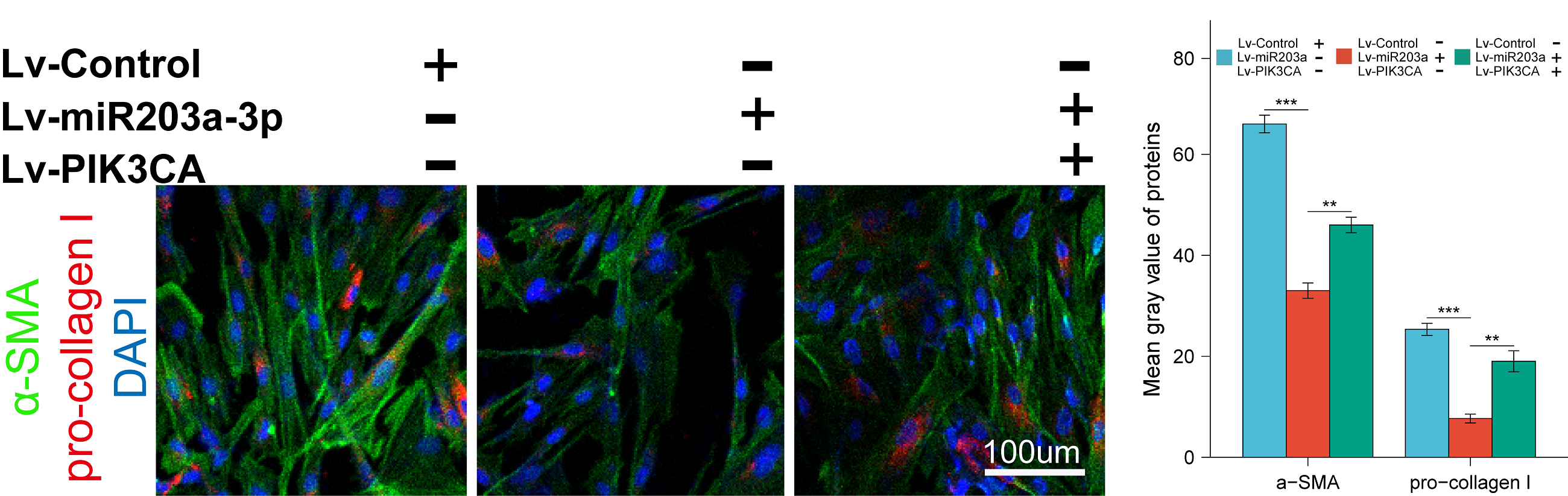


**Figure S5.** Representative images of immunofluorescence and quantitative analysis of α-SMA and pro-collagen I (n=3), Scale bar: 100μm. ***p* < 0.01; ****p* < 0.001
