# Supplementary material for: Inhibition of phosphatidylinositol 3-kinase catalytic subunit alpha by miR-203a-3p reduces hypertrophic scar formation via phosphatidylinositol 3-kinase/AKT/mTOR signaling pathway
Source: Burns Trauma. 2024 Jan 2;12:tkad048. doi: 10.1093/burnst/tkad048 (PMC10762504; doi:10.1093/burnst/tkad048)
Supplement: Table_S1_tkad048 [file table_s1_tkad048.docx]

**Table S1. Patients’ information**

| **Patient numbers** | **Gender** | **Age** | **Location** |
| --- | --- | --- | --- |
| 1 | Female | 25 | face |
| 2 | Male | 24 | right hand |
| 3 | Male | 24 | left hand |
| 4 | Female | 27 | neck |
| 5 | Male | 24 | neck |
| 6 | Female | 21 | left arm |
| 7 | Male | 20 | left arm |
| 8 | Male | 18 | shoulder |
