# Supplementary material for: Inhibition of phosphatidylinositol 3-kinase catalytic subunit alpha by miR-203a-3p reduces hypertrophic scar formation via phosphatidylinositol 3-kinase/AKT/mTOR signaling pathway
Source: Burns Trauma. 2024 Jan 2;12:tkad048. doi: 10.1093/burnst/tkad048 (PMC10762504; doi:10.1093/burnst/tkad048)
Supplement: Table_S2_tkad048 [file table_s2_tkad048.docx]

**Table S2. Information of primary antibody**

| **Name** | **Dilution in WB** | **Dilution in IHC** | **Dilution in IF/ICC** | **Manufacturer** | **Cat. No.** |
| --- | --- | --- | --- | --- | --- |
| PIK3CA | 1:1000 |  | 1:200 | Abcam | ab40776 |
| PIK3CA |  | 1:2000 |  | Servicebio | GB11769 |
| Col1A1 | 1:1000 |  | 1:200 | CST | #66948 |
| Col3 | 1:1000 |  |  | Abcam | ab184993 |
| α-SMA | 1:1000 |  | 1:200 | Abcam | Ab124964 |
| p-mTOR | 1:1000 |  |  | Abcam | ab109268 |
| mTOR | 1:1000 |  |  | Abcam | ab134903 |
| p-P85 | 1:1000 |  |  | Abcam | ab182651 |
| P85 | 1:1000 |  |  | Abcam | ab191606 |
| p-AKT | 1:1000 |  |  | Abcam | ab192623 |
| AKT | 1:1000 |  |  | Abcam | ab179463 |
| GAPDH | 1:10000 |  |  | CST | #5174 |
| pro-collagen I |  |  | 1:200 | DSHB | M-38 |
| Ki67 |  |  | 1:1000 | Abcam | Ab15580 |
| F-actin |  |  | 1:20 | Invitrogen | R37110 |
