# Supplementary material for: Inhibition of phosphatidylinositol 3-kinase catalytic subunit alpha by miR-203a-3p reduces hypertrophic scar formation via phosphatidylinositol 3-kinase/AKT/mTOR signaling pathway
Source: Burns Trauma. 2024 Jan 2;12:tkad048. doi: 10.1093/burnst/tkad048 (PMC10762504; doi:10.1093/burnst/tkad048)
Supplement: Table_S4_tkad048 [file table_s4_tkad048.docx]

**Table S4. miRNA primer sequences**

| **miRNA Name** | **Forward Sequence** | **Reverse Sequence** |
| --- | --- | --- |
| miR-203a-3p | ACACTCCAGCTGGGGTGAAATGTTTAGGAC | CTCAACTGGTGTCGTGGAGTCGGCAATTCAGTTGAGCTAGTGGT |
| U6 | CTCGCTTCGGCAGCACA | AACGCTTCACGAATTTGCGT |
